# Joint assessment of metabolic and functional indices for coronary burden: a real-world study in patients undergoing angiography for chest pain

**DOI:** 10.3389/fcvm.2026.1835613

**Published:** 2026-08-13

**Authors:** Xingxing Che, Mengting An, Wenkai Liu, Zhongjian Hao, Ting Gao, Gang Qin

**Affiliations:** Department of Cardiology, The First Hospital of Shanxi Medical University, Taiyuan, China

**Keywords:** coronary heart disease, Gensini score, left ventricular ejection fraction, risk stratification, triglyceride-glucose index

## Abstract

**Objective:**

To investigate the associations of composite glucose–lipid metabolic indices and echocardiographic parameters with coronary heart disease (CHD), and to determine whether integrating metabolic burden and cardiac function could improve pre-angiographic risk stratification and reflect coronary lesion severity.

**Methods:**

This cross-sectional study consecutively enrolled 1,061 patients with chest pain who underwent coronary angiography at the First Hospital of Shanxi Medical University. CHD was defined as ≥50% stenosis in at least one major epicardial coronary artery. Composite metabolic indices, including the triglyceride-glucose index (TyG), TyG-BMI, METS-IR, atherogenic index of plasma, remnant cholesterol, and lipoprotein combine index, were evaluated together with echocardiographic parameters. Multivariable logistic regression was used to identify independent correlates of CHD. Tertile analysis and restricted cubic spline models were applied to assess dose-response patterns and potential non-linearity. ROC curves were used to compare discriminatory ability, and Spearman's correlation together with the Gensini score was used to evaluate coronary atherosclerotic burden.

**Results:**

Among 1,061 participants, 522 had CHD. After multivariable adjustment, TyG (OR = 1.588, 95% CI: 1.269–1.995) and left ventricular ejection fraction (LVEF; OR = 0.958, 95% CI: 0.943–0.973) remained the two strongest independent indicators of CHD (both *P* < 0.001). TyG showed an approximately linear positive association with CHD risk, whereas LVEF showed a non-linear inverse association. Adding both measures to the base clinical model increased the AUC from 0.655 to 0.694, with decision-curve analysis indicating a modest incremental net benefit, although overall discrimination remained moderate. The high-TyG/low-LVEF group had the highest estimated CHD probability and greatest angiographic disease burden, as quantified by the Gensini score.

**Conclusion:**

TyG and LVEF are routinely available, complementary measures of metabolic burden and systolic function. Both were independently associated with angiographically defined CHD, while the high-TyG/low-LVEF phenotype showed greater coronary burden. Their joint assessment modestly improved discrimination and may inform exploratory pre-angiographic evaluation in chest-pain patients.

## Introduction

Coronary heart disease (CHD) remains a major impediment to global public health. Data from the Global Burden of Disease study indicate a sustained rise in cardiovascular disease burden, with disability adjusted life years reaching approximately 437 million in 2023, representing about a 1.4 fold increase compared with 1990 when the estimate was 320 million, a pattern increasingly driven by metabolic risk factors (1). The development of CHD is typically covert and slowly progressive, with substantial atherosclerotic accumulation and microvascular dysfunction often preceding overt symptoms such as angina or acute coronary syndromes (2, 3). Although coronary angiography (CAG) is the definitive reference standard for assessing luminal stenosis, its invasiveness, cost, and procedure related risks limit its suitability for population wide screening or routine surveillance in low risk individuals (4, 5). Therefore, there is a pressing need for a non invasive and cost effective risk stratification approach based on routinely available clinical measures to enable earlier identification of high risk individuals and timely prevention of disease progression.

Systemic metabolic dysregulation is a fundamental driver of CHD, with insulin resistance serving as a key pathophysiological link between disturbed glucose lipid homeostasis and atherosclerotic progression (6, 7). Although fasting glucose and LDL cholesterol are routinely used in clinical practice, they may not adequately reflect the overall metabolic burden under insulin resistance (8). This shortfall may partly account for the residual cardiovascular risk observed even among individuals who achieve guideline recommended lipid targets. In this setting, the triglyceride glucose (TyG) index has gained attention as a pragmatic surrogate marker of insulin resistance (9, 10). Derived from two widely available fasting biochemical measures and requiring only simple calculation, TyG is readily applicable in routine care (9). Growing evidence suggests that higher TyG levels are associated with greater coronary lesion burden and poorer prognosis, supporting its role as an upstream indicator of metabolic stress on the vasculature (11). Beyond TyG, several related composite metrics, including TyG BMI, METS IR, AIP, RC, LCI, and the Castelli risk indices, have been developed to characterise lipid patterns, residual cholesterol exposure, and atherogenic propensity from complementary dimensions, offering additional candidates for more granular risk stratification (12, 13).

In parallel with upstream metabolic stress, changes in cardiac structure and function constitute key downstream manifestations along the CHD continuum. Transthoracic echocardiography is a cornerstone non-invasive modality in routine practice, and left ventricular ejection fraction (LVEF) remains one of the most widely used indicators of systolic performance (14). Beyond capturing myocardial dysfunction secondary to ischemia, LVEF also reflects overall cardiac functional reserve and vulnerability to adverse remodeling (15). Nevertheless, existing evidence is often generated within a compartmentalized framework, in which metabolic indices and echocardiographic phenotypes are examined separately. Systematic investigations that integrate metabolic drivers with cardiac functional phenotypes remain limited (16). Considering that metabolic toxicity, including glucolipotoxicity, can directly promote myocardial injury and remodeling, an assessment strategy combining these domains is both biologically plausible and potentially more informative for clinical risk profiling (17).

To address these gaps, we conducted a retrospective cross-sectional study using real-world data from patients with chest pain who underwent coronary angiography. Logistic regression models were fitted to evaluate the associations of glucose-lipid metabolic indices, including TyG, TyG-BMI, METS-IR, AIP, RC, and LCI, and echocardiographic parameters, including LADI and LVEF, with angiographically defined CHD. Restricted cubic spline analyses were subsequently used to characterize dose-response relationships and assess potential nonlinearity. Associations with coronary atherosclerotic burden were further examined using the Gensini score. Finally, we assessed whether incorporating TyG and LVEF into a parsimonious clinical model improved discrimination and provided incremental net benefit for identifying CHD.

## Method

### Study population and eligibility criteria

This cross-sectional study enrolled 1,061 consecutive patients presenting with chest pain at the First Hospital of Shanxi Medical University between 5 September 2024 and 17 June 2025 who underwent coronary angiography (Supplementary Figure S1). CHD was defined angiographically as ≥50% luminal diameter stenosis in at least one major epicardial coronary artery (left main, left anterior descending, left circumflex, right coronary artery, or their major branches). The extent and severity of coronary artery disease were assessed using the Gensini score (18). Specifically, each lesion was graded by stenosis severity (25%=1, 50%=2, 75%=4, 90%=8, 99%=16, and total occlusion=32) and then weighted by its anatomical location (e.g., left main ×5; proximal LAD/LCX ×2.5; mid-LAD ×1.5; RCA and distal LAD/posterior descending/obtuse marginal branches ×1.0; other branches ×0.5). The overall Gensini score was calculated as the sum of all weighted segment scores, with higher totals reflecting more extensive and severe disease.

Eligible participants were adults (>18 years) undergoing their first coronary angiography, with complete clinical information and written informed consent. Patients were excluded if they had a previous diagnosis of CHD or had undergone coronary revascularization, including PCI or CABG. Individuals with other confirmed cardiac disorders, including rheumatic heart disease, valvular heart disease, cardiomyopathy, or advanced heart failure, were also excluded. In addition, those with severe end-organ failure were ineligible, including active malignancy, end-stage kidney failure, or decompensated liver cirrhosis. This study was approved by the Ethics Committee of the First Hospital of Shanxi Medical University.

### Clinical data collection and index calculation

Demographic characteristics and medical history were retrospectively obtained from the electronic medical record system and included as covariates, encompassing sex, age, type 2 diabetes mellitus (T2DM), hypertension (HTN), alcohol use, and smoking status. Fasting biochemical measurements collected during hospitalization included fasting plasma glucose (FPG), total cholesterol (TC), triglycerides (TG), low-density lipoprotein cholesterol (LDL-C), and high-density lipoprotein cholesterol (HDL-C). Using these variables, glucose-lipid metabolism composite indices were computed according to standard equations: Body mass index (BMI) = weight (kg)/height (m)^2^; TyG index = ln[TG (mg/dL) × FPG (mg/dL)/2]; TyG-BMI = TyG×BMI; METS-IR = (ln[(2 × FPG (mg/dL)) + TG (mg/dL)] × BMI)/ln[HDL-C (mg/dL)]; atherogenic index of plasma (AIP) = log₁₀(TG/HDL-C); residual cholesterol (RC) = TC−LDL-C−HDL-C; lipoprotein composite index (LCI) = TC × TG × LDL-C/HDL-C; Castelli risk indices CRI-I = TC/HDL-C and CRI-II = LDL-C/HDL-C; and atherosclerosis coefficient (AC) = (TC−HDL-C)/HDL-C. Cardiac structure and function parameters were retrieved from contemporaneous examinations, including left atrial diameter (LAD) and left ventricular ejection fraction (LVEF) from echocardiography, and angiographic disease burden quantified by the Gensini score. The left atrial diameter index (LADI) was calculated as LAD/body surface area (BSA), with BSA = √[height (cm) × weight (kg)/3600] (19).

### Statistical analysis

All statistical analyses were conducted in R (version 4.4.0). The distribution of continuous variables was examined using the Shapiro–Wilk test. Data approximating a normal distribution are reported as mean ± standard deviation (SD), whereas skewed data are summarised as median with interquartile range [M (IQR)]. Categorical variables are presented as counts and percentages. Between-group comparisons were performed using an independent-samples t test for normally distributed continuous variables and the Mann–Whitney U test for non-normally distributed variables. Categorical variables were compared using the *χ*^2^ test, with Fisher's exact test applied when appropriate. All analyses were two-sided, and a *P* value < 0.05 was considered statistically significant.

Associations of glucose-lipid metabolic composite indices and cardiac structural/functional parameters with CHD were examined using logistic regression. Effect estimates are reported as odds ratios (ORs) with 95% confidence intervals (CIs). To account for potential confounding, four models were specified *a priori*: Model 0 (unadjusted); Model 1 adjusted for age, sex, and BMI; Model 2 additionally adjusted for lifestyle factors (smoking and alcohol consumption); and Model 3 further adjusted for comorbidities, including hypertension (HTN), Hyperlipidaemia (HLP) and type 2 diabetes mellitus (T2DM). Each index was also categorised into quantiles to compare CHD risk across strata and evaluate linear trends. Finally, restricted cubic spline (RCS) modelling was applied to characterise dose-response patterns and to test for potential non-linear associations between the composite indices and CHD.

Stratified subgroup analyses were performed by age, sex, BMI, smoking and alcohol use, and the presence of hypertension and diabetes to examine the robustness of the associations between the composite indices and CHD across clinically relevant strata. Discriminative performance was evaluated using receiver operating characteristic (ROC) curves, with the area under the curve (AUC) used to compare predictive ability among indices. The prespecified base clinical model included age, sex, body mass index, smoking status, alcohol consumption, and hypertension; the augmented model further incorporated the triglyceride–glucose index and left ventricular ejection fraction. Model discrimination was quantified by the area under the receiver operating characteristic curve, and between-model differences were evaluated using DeLong's test. Exploratory clinical utility was assessed by decision-curve analysis using out-of-fold probabilities generated for each individual through 20 repetitions of stratified 10-fold cross-validation. Across a range of threshold probabilities, net benefit was compared among the base model, augmented model, treat-all strategy, and treat-none strategy. To further characterise the link between composite indices and angiographic disease burden, Spearman correlation and multivariable linear regression were applied to examine associations between Gensini scores and each composite index among participants with assessable coronary lesions. All analyses were two-sided, and a *P* value < 0.05 was considered statistically significant.

## Results

### Baseline characteristics between the CHD and non-CHD groups

A total of 1,061 patients presenting with chest pain were included (505 men and 556 women), among whom 522 were diagnosed with CHD. The mean age was 64 years in the CHD group and 60 years in the non-CHD group. Baseline demographic and clinical characteristics are summarised in Table 1. Compared with the non-CHD cohort, patients with CHD were older and had higher fasting glucose levels, TyG index values, and LADI, but lower LVEF, and they more frequently had hypertension and type 2 diabetes mellitus (*P* < 0.05).

**Table 1:** Baseline characteristics of participants with and without coronary heart disease (CHD).

Characteristic	Non-CHD	CHD	*P*-value
Population	539	522	
Age	60 (53.5, 67)	64 (58, 71)	<0.001
Sex			0.619
Female	287 (53.2%)	269 (51.5%)	
Male	252 (46.8%)	253 (48.5%)	
BMI	25.39 (23.06, 27.95)	24.5 (22.77, 27.05)	0.003
Smoking			0.616
No	362 (67.2%)	342 (65.5%)	
Yes	177 (32.8%)	180 (34.5%)	
Alcohol			0.605
No	411 (76.3%)	406 (77.8%)	
Yes	128 (23.7%)	116 (22.2%)	
HTN			<0.001
No	262 (48.6%)	170 (32.6%)	
Yes	277 (51.4%)	352 (67.4%)	
T2DM			<0.001
No	438 (81.3%)	330 (63.2%)	
Yes	101 (18.7%)	192 (36.8%)	
HLP			0.359
No	275 (51.0%)	282 (54.0%)	
Yes	264 (49.0%)	240 (46.0%)	
HDL	1.14 (0.96, 1.3)	1.08 (0.92, 1.27)	0.003
FPG	5.15 (4.75, 5.92)	5.72 (5, 7.01)	<0.001
TC	4.22 (3.5, 4.92)	3.77 (3.07, 4.68)	<0.001
TG	1.39 (1.03, 1.95)	1.4 (1.02, 1.94)	0.81
LDL	2.6 (2.05, 3.14)	2.25 (1.8, 2.93)	<0.001
LVEF	64 (60, 68)	62 (56, 66)	<0.001
LAD	3.6 (3.3, 3.9)	3.6 (3.3, 3.9)	0.086
Gensini	1 (0, 4)	41 (21, 72)	<0.001
AC	2.68 (2.11, 3.31)	2.49 (1.91, 3.3)	0.036
AIP	0.09 (−0.07, 0.28)	0.13 (−0.06, 0.29)	0.132
CRI_I	3.68 (3.11, 4.31)	3.49 (2.91, 4.3)	0.036
CRI_II	2.26 (1.85, 2.79)	2.12 (1.66, 2.77)	0.011
LCI	13.19 (7.56, 23.48)	11.38 (5.77, 21.39)	0.008
RC	0.42 (0.29, 0.6)	0.39 (0.26, 0.58)	0.091
TyG	8.72 (8.35, 9.07)	8.79 (8.45, 9.2)	0.011
TyG_BMI	221.25 (197.2, 249.88)	217.78 (198.01, 240.66)	0.143
METS_IR	38.92 (34.55, 44.15)	39 (34.72, 43.45)	0.805
LADI	2.02 (1.84, 2.17)	2.08 (1.9, 2.26)	<0.001

BMI, body mass index; HTN, hypertension; T2DM, type 2 diabetes mellitus; HLP, hyperlipidaemia; HDL, high-density lipoprotein cholesterol; FPG, fasting plasma glucose; TC, total cholesterol; TG, triglycerides; LDL, low-density lipoprotein cholesterol; LVEF, left ventricular ejection fraction; LAD, left atrial diameter; LADI, left atrial diameter index; AC, atherosclerosis coefficient; AIP, atherogenic index of plasma; CRI-I/CRI-II, Castelli risk indices I/II; LCI, lipoprotein composite index; RC, residual cholesterol; TyG, triglyceride–glucose index; TyG-BMI, TyG multiplied by BMI. *P* values <0.05 were considered statistically significant.

### Univariate and multivariable logistic regression results for CHD

Univariate logistic regression identified LVEF, TyG, LADI, and CRI_II as variables significantly associated with CHD (Table 2). After inclusion in multivariable models with full adjustment for covariates, TyG remained independently associated with higher odds of CHD (OR = 1.588, 95% CI: 1.269–1.995, *P* < 0.001). AIP was also positively associated with CHD (OR = 1.713, 95% CI: 1.004–2.937, *P* = 0.049), although the strength of evidence was borderline. In contrast, LVEF showed an independent inverse association with CHD (OR = 0.958, 95% CI: 0.943–0.973, *P* < 0.001), consistent with a protective effect. Notably, the associations for LADI and CRI_II were attenuated and were no longer statistically significant after covariate adjustment.

**Table 2:** Univariate and multivariable logistic regression models for the associations of glucose–lipid metabolic composite indices and cardiac structural/functional parameters with CHD.

	Model 0		Model 1		Model 2		Model 3	
Variables	OR (95% CI)	*P* value	OR (95% CI)	*P* value	OR (95% CI)	*P* value	OR (95% CI)	*P* value
LVEF	0.961 (0.947–0.975)	<0.001	0.958 (0.943–0.972)	<0.001	0.958 (0.943–0.973)	<0.001	0.958 (0.943–0.973)	<0.001
TyG	1.329 (1.084–1.634)	0.007	1.651 (1.324–2.068)	<0.001	1.652 (1.324–2.069)	<0.001	1.588 (1.269–1.995)	<0.001
TyG_BMI	0.997 (0.994–1.001)	0.115	1.001 (0.998–1.004)	0.578	1.001 (0.998–1.004)	0.591	0.999 (0.995–1.003)	0.596
METS_IR	0.996 (0.979–1.012)	0.605	1.015 (0.997–1.033)	0.106	1.014 (0.996–1.033)	0.119	1.005 (0.986–1.024)	0.592
AIP	1.230 (0.763–1.986)	0.395	2.159 (1.289–3.634)	0.004	2.112 (1.260–3.559)	0.005	1.713 (1.004–2.937)	0.049
RC	0.760 (0.511–1.115)	0.165	0.922 (0.614–1.372)	0.693	0.920 (0.612–1.368)	0.681	0.913 (0.602–1.374)	0.663
LCI	0.995 (0.989–1.001)	0.111	0.999 (0.993–1.005)	0.829	0.999 (0.993–1.005)	0.828	0.998 (0.992–1.004)	0.588
LADI	2.147 (1.408–3.299)	<0.001	1.407 (0.879–2.261)	0.156	1.379 (0.860–2.220)	0.184	1.227 (0.757–1.993)	0.408
CRI_I	0.892 (0.784–1.013)	0.081	0.983 (0.860–1.124)	0.806	0.982 (0.858–1.122)	0.786	0.970 (0.846–1.113)	0.667
CRI_II	0.846 (0.715–0.999)	0.049	0.952 (0.800–1.132)	0.576	0.951 (0.799–1.132)	0.572	0.945 (0.791–1.129)	0.534
AC	0.892 (0.784–1.013)	0.081	0.983 (0.860–1.124)	0.806	0.982 (0.858–1.122)	0.786	0.970 (0.846–1.113)	0.667

Effect estimates are presented as odds ratios (ORs) with 95% confidence intervals (CIs). Four models were prespecified: Model 0, unadjusted; Model 1, adjusted for age, sex, and BMI; Model 2, additionally adjusted for smoking and alcohol consumption; Model 3, further adjusted for hypertension (HTN), Hyperlipidaemia (HLP) and type 2 diabetes mellitus (T2DM). *P* values are two-sided, and *P* < 0.05 was considered statistically significant. LVEF, left ventricular ejection fraction; TyG, triglyceride–glucose index; TyG-BMI, TyG multiplied by BMI; METS-IR, metabolic score for insulin resistance; AIP, atherogenic index of plasma; RC, residual cholesterol; LCI, lipoprotein composite index; LADI, left atrial diameter index; CRI-I/CRI-II, Castelli risk indices I/II; AC, atherosclerosis coefficient.

### Dose-response associations of TyG and LVEF with CHD

After adjustment for all potential confounders, logistic regression demonstrated a significant positive association between the TyG index and CHD. Relative to the low TyG group, the odds of CHD were higher in the moderate TyG group (OR = 1.388, 95% CI: 1.012–1.906, *P* = 0.042) and increased further in the high TyG group (OR = 1.854, 95% CI: 1.345–2.563, *P* < 0.001). In contrast, LVEF was inversely associated with CHD. Compared with the low LVEF group, participants in the moderate LVEF group and the high LVEF group showed lower odds of CHD (OR = 0.570, 95% CI: 0.418–0.776, *P* < 0.001) and (OR = 0.454, 95% CI: 0.327–0.627, *P* < 0.001), respectively (Table 3).

**Table 3:** Tertile-based associations of LVEF and TyG with CHD in multivariable logistic regression models.

		Model 1		Model 2		Model 3	
Variables		OR (95% CI)	*P* value	OR (95% CI)	*P* value	OR (95% CI)	*P* value
LVEF	T1	1.00 (Reference)	-	1.00 (Reference)	-	1.00 (Reference)	-
LVEF	T2	0.615 (0.456–0.828)	0.001	0.614 (0.455–0.828)	0.001	0.570 (0.418–0.776)	<0.001
LVEF	T3	0.449 (0.326–0.615)	<0.001	0.454 (0.330–0.623)	<0.001	0.454 (0.327–0.627)	<0.001
TyG	T1	1.00 (Reference)	-	1.00 (Reference)	-	1.00 (Reference)	-
TyG	T2	1.388 (1.017–1.899)	0.04	1.384 (1.013–1.895)	0.042	1.388 (1.012–1.906)	0.042
TyG	T3	1.950 (1.422–2.686)	<0.001	1.955 (1.424–2.694)	<0.001	1.854 (1.345–2.563)	<0.001

LVEF and TyG were categorised into tertiles (T1–T3), with T1 as the reference group. Effect estimates are presented as odds ratios (ORs) with 95% confidence intervals (CIs). Model 1 adjusted for age, sex, and BMI; Model 2 additionally adjusted for smoking and alcohol consumption; Model 3 further adjusted for hypertension (HTN), Hyperlipidaemia (HLP) and type 2 diabetes mellitus (T2DM). *P* values are two-sided, and *P* < 0.05 was considered statistically significant. LVEF, left ventricular ejection fraction; TyG, triglyceride–glucose index.

To further assess the joint effects of metabolic burden and cardiac function on CHD, we cross-classified TyG and LVEF into tertiles, yielding nine combined subgroups. The “high TyG + low LVEF” category was set as the reference (Table 4 and Supplementary Table S1). Compared with this reference, groups characterised by higher LVEF consistently showed lower odds of CHD. Even among participants with high TyG, those with high LVEF had substantially reduced CHD odds (OR = 0.418, *P* = 0.001). Similar or greater reductions were observed when high LVEF co-occurred with moderate or low TyG (OR = 0.345, *P* < 0.001; and OR = 0.319, *P* < 0.001, respectively). In addition, the low TyG + moderate LVEF group also exhibited markedly lower CHD odds (OR = 0.255, *P* < 0.001). By contrast, within the low LVEF stratum, reductions associated with lower TyG were smaller and did not reach statistical significance in some comparisons (*P* > 0.05). Overall, these results suggest that higher LVEF may partially attenuate the CHD odds associated with elevated TyG, whereas the combination of high TyG and low LVEF corresponds to a comparatively higher CHD risk profile.

**Table 4:** Joint associations of TyG and LVEF tertile combinations with CHD in multivariable logistic regression models.

	Model 1		Model 2		Model 3	
Variables	OR (95% CI)	*P* value	OR (95% CI)	*P* value	OR (95% CI)	*P* value
High TyG + Low LVEF	1.00 (Reference)	-	1.00 (Reference)	-	1.00 (Reference)	-
High TyG + High LVEF	0.422 (0.250–0.706)	0.001	0.428 (0.254–0.716)	0.001	0.418 (0.246–0.703)	0.001
High TyG + Mid LVEF	0.690 (0.399–1.192)	0.184	0.694 (0.400–1.201)	0.191	0.676 (0.388–1.178)	0.167
Low TyG + High LVEF	0.304 (0.182–0.503)	<0.001	0.311 (0.186–0.514)	<0.001	0.319 (0.189–0.531)	<0.001
Low TyG + Low LVEF	0.529 (0.312–0.890)	0.017	0.528 (0.312–0.890)	0.017	0.577 (0.338–0.979)	0.042
Low TyG + Mid LVEF	0.257 (0.147–0.443)	<0.001	0.252 (0.144–0.435)	<0.001	0.255 (0.144–0.444)	<0.001
Mid TyG + High LVEF	0.324 (0.193–0.537)	<0.001	0.321 (0.192–0.533)	<0.001	0.345 (0.204–0.577)	<0.001
Mid TyG + Low LVEF	0.685 (0.405–1.155)	0.156	0.683 (0.404–1.153)	0.154	0.734 (0.431–1.249)	0.255
Mid TyG + Mid LVEF	0.521 (0.307–0.878)	0.015	0.527 (0.310–0.890)	0.017	0.514 (0.301–0.874)	0.014

TyG and LVEF were each categorised into tertiles and cross-classified into nine combined groups. “High TyG + Low LVEF” was used as the reference category. Effect estimates are presented as odds ratios (ORs) with 95% confidence intervals (CIs). Model 1 adjusted for age, sex, and BMI; Model 2 additionally adjusted for smoking and alcohol consumption; Model 3 further adjusted for hypertension (HTN) and type 2 diabetes mellitus (T2DM). *P* values are two-sided, and *P* < 0.05 was considered statistically significant. TyG, triglyceride–glucose index; LVEF, left ventricular ejection fraction.

**Figure 1:**
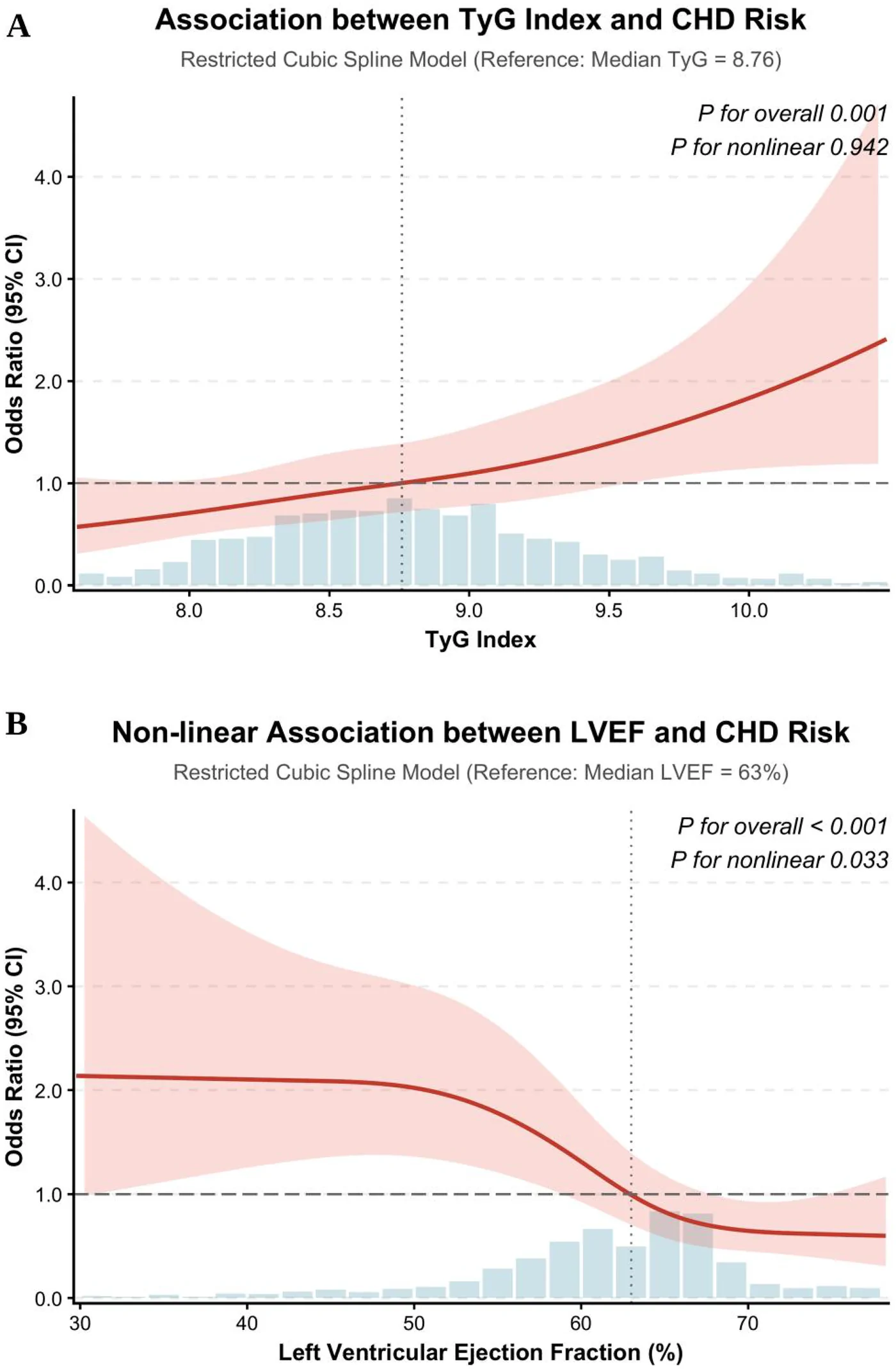
Restricted cubic spline (RCS) analyses of TyG index and LVEF in relation to CHD risk. **(A)** Dose-response association between the TyG index and CHD risk estimated using an RCS logistic regression model. **(B)** Non-linear association between left ventricular ejection fraction (LVEF) and CHD risk based on an RCS model. Histograms depict the distribution of TyG and LVEF in the study population. *P* values for overall association and non-linearity are shown in each panel. Adjustment was made for all covariates, including age, sex, and BMI; smoking status and alcohol consumption; and comorbidities of hypertension (HTN), hyperlipidaemia (HLP), and type 2 diabetes mellitus (T2DM).

RCS modelling further indicated an approximately linear dose-response relationship between TyG and CHD (P for non-linear=0.942), whereas the association between LVEF and CHD was non-linear (P for non-linear=0.033) (Figure 1).

### Subgroup analyses of CHD risk

**Figure 2:**
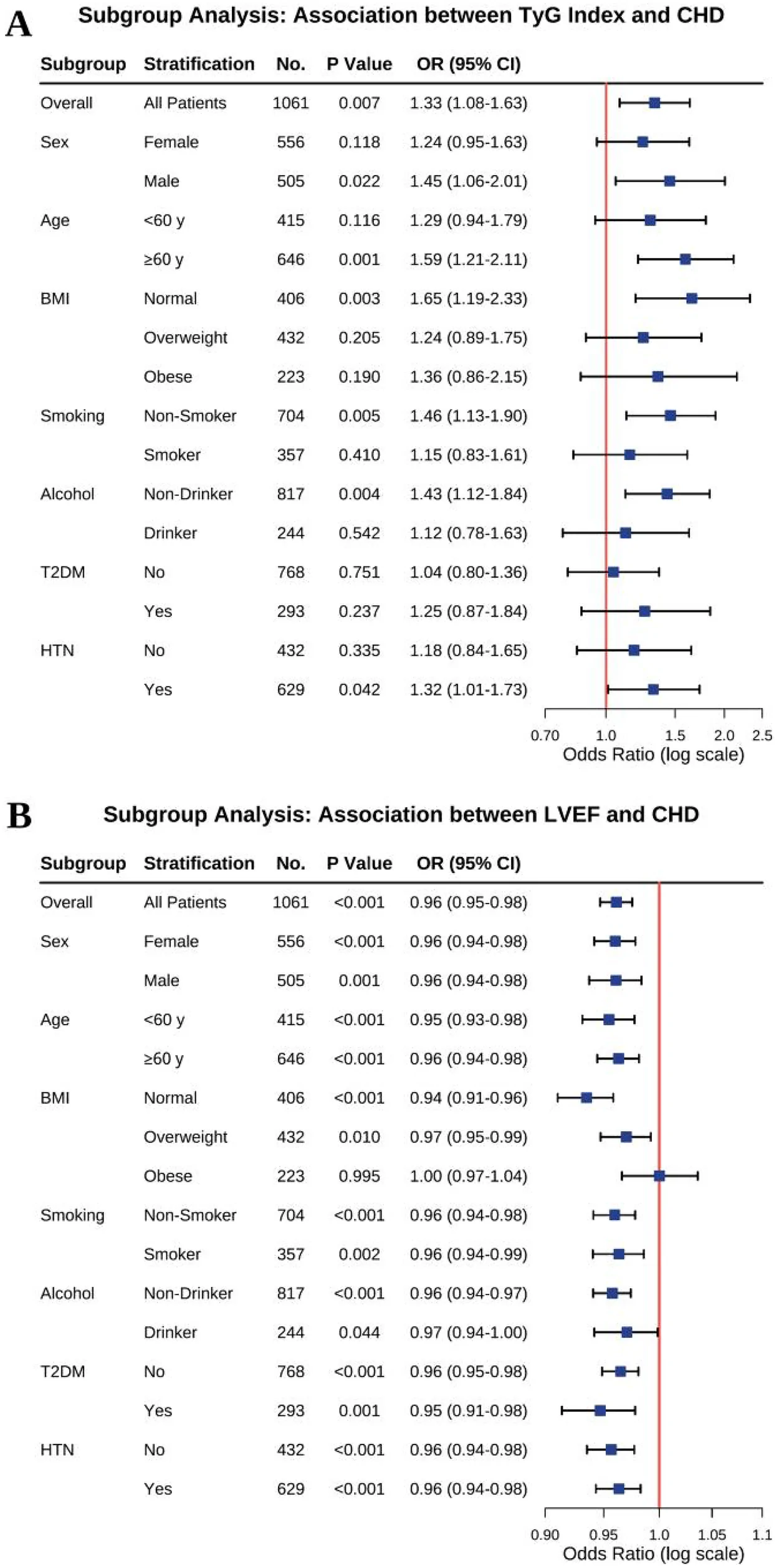
Subgroup analyses of the associations between TyG, LVEF, and CHD. Forest plots present odds ratios (ORs) with 95% confidence intervals (CIs) for CHD across predefined subgroups stratified by sex, age, BMI category, smoking status, alcohol consumption, type 2 diabetes mellitus (T2DM), and hypertension (HTN). **(A)** Association between the TyG index and CHD within each subgroup. **(B)** Association between left ventricular ejection fraction (LVEF) and CHD within each subgroup. The vertical reference line indicates OR = 1.0, and estimates are displayed on a logarithmic scale.

To examine whether the associations of LVEF and TyG with CHD were consistent across clinically relevant strata, we performed stratified subgroup analyses by sex, age (60-year cutoff), BMI category (normal vs. overweight), lifestyle factors (smoking and alcohol use), and comorbidities (hypertension and diabetes) (Figure 2). LVEF showed a stable association with CHD in every subgroup evaluated, indicating robust and broadly consistent effects (Supplementary Table S2). By contrast, the TyG index displayed substantial heterogeneity across strata. Statistically significant associations with CHD were observed mainly among men, participants aged >60 years, non-smokers, non-drinkers, and those with hypertension (Supplementary Table S3).

### ROC curve analysis of TyG, LVEF, and the combined model

**Figure 3:**
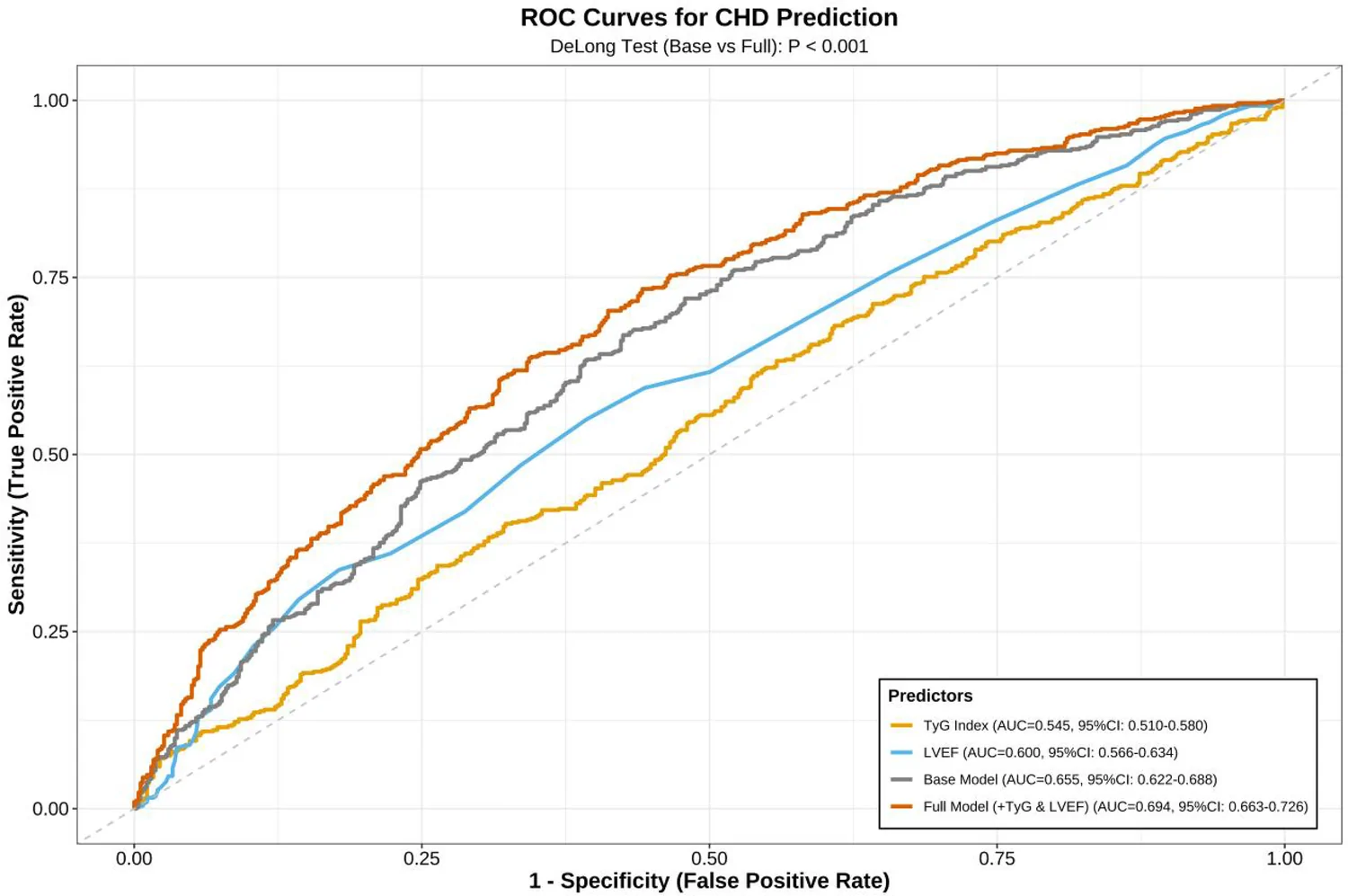
ROC curve analysis for CHD discrimination using TyG, LVEF, the base clinical model, and the TyG-LVEF-augmented model. Receiver operating characteristic (ROC) curves were constructed to compare the discriminatory performance of the TyG index alone, LVEF alone, a base clinical model, and an augmented model for identifying CHD. The base clinical model included age, sex, BMI, smoking status, alcohol consumption, and hypertension (HTN); the augmented model additionally included TyG and LVEF. The area under the ROC curve (AUC) with 95% confidence intervals (CIs) is reported for each curve. The diagonal dashed line denotes no-discrimination (AUC=0.50). The AUC increased from 0.655 for the base model to 0.694 for the augmented model (DeLong *P* < 0.001).

**Figure 4:**
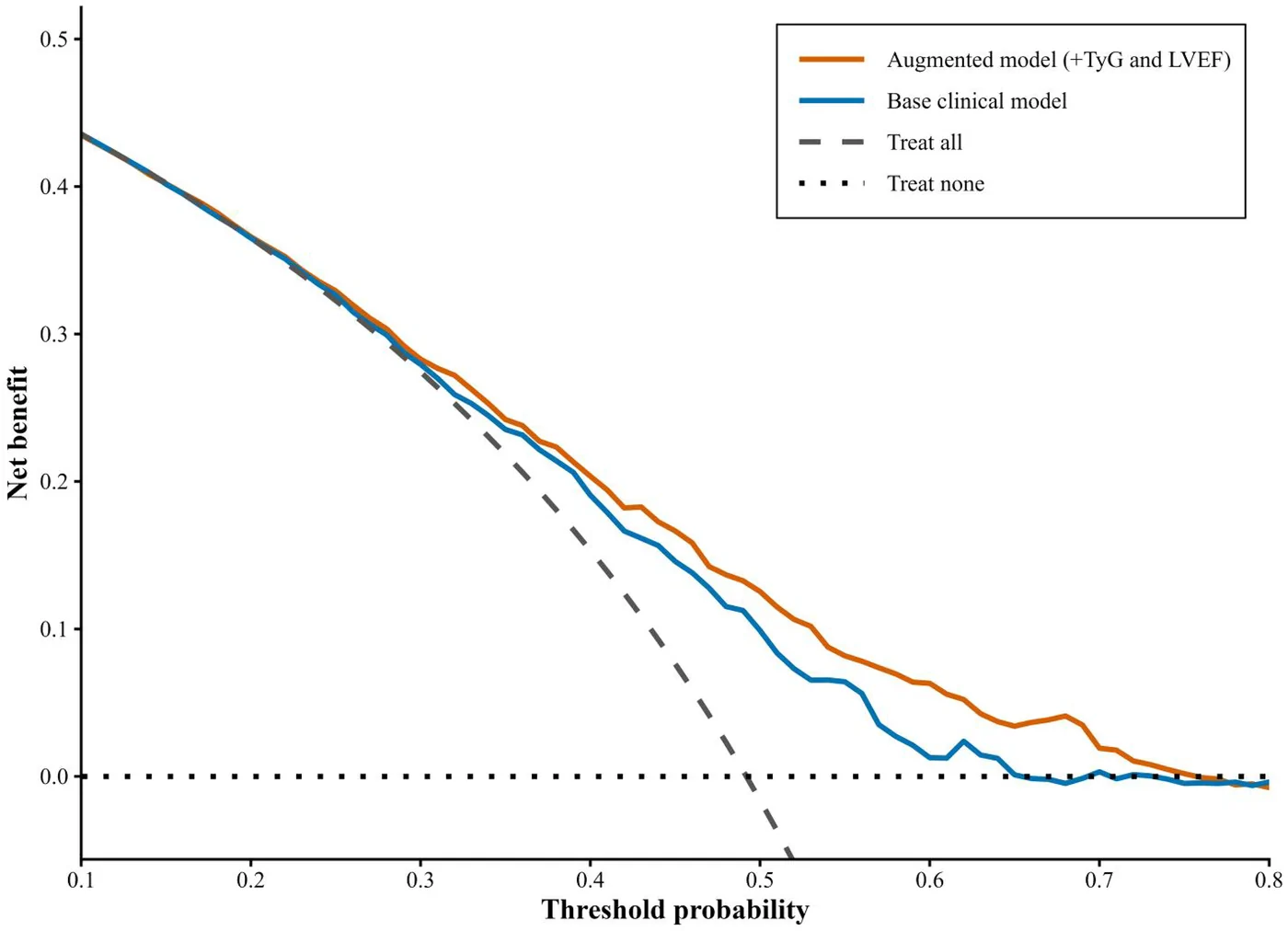
Decision-curve analysis of the base and TyG-LVEF-augmented models for identifying angiographically confirmed CHD. Net-benefit curves were calculated using individual predicted probabilities from 20 repeats of stratified 10-fold cross-validation. The base clinical model included age, sex, BMI, smoking status, alcohol consumption, and hypertension; the augmented model additionally included TyG and LVEF. The augmented model provided greater net benefit than the base model between threshold probabilities of 0.20 and 0.75. Treat-all and treat-none strategies are shown for reference. This internally validated analysis remains exploratory and does not establish external transportability.

ROC curve analysis indicated that the discriminative performance of single markers for CHD was generally modest (Figure 3 and Supplementary Table S4). The TyG index showed relatively limited predictive ability (AUC=0.545, 95% CI: 0.510–0.580, *P* = 0.011), whereas LVEF performed somewhat better, although its AUC remained moderate (AUC=0.600, 95% CI: 0.566–0.634, *P* < 0.001). The base clinical model, which included age, sex, body mass index, smoking status, alcohol consumption, and hypertension, yielded an AUC of 0.655 (95% CI, 0.622–0.688). Adding the triglyceride–glucose index and left ventricular ejection fraction increased the AUC to 0.694 (95% CI, 0.663–0.726), an absolute gain of 0.039 (*P* < 0.001 by DeLong's test). Despite this statistically significant increase, the augmented model showed moderate discrimination overall. Youden index analysis yielded thresholds of TyG > 8.963 and LVEF < 58.5%. Given the modest AUCs and absence of external validation, these thresholds should be regarded as exploratory. Across 20 repetitions of stratified 10-fold cross-validation, the base and augmented models yielded AUCs of 0.645 and 0.683, respectively, an absolute difference of 0.038 (*P* < 0.001 by DeLong's test). Decision-curve analysis showed that the augmented model provided greater net benefit than the base model across threshold probabilities from 0.20 to 0.75 (Figure 4). The absolute gain was nevertheless modest, with a median net-benefit difference of 0.0160.

### Association between composite indicators and severity of coronary artery lesions

**Figure 5:**
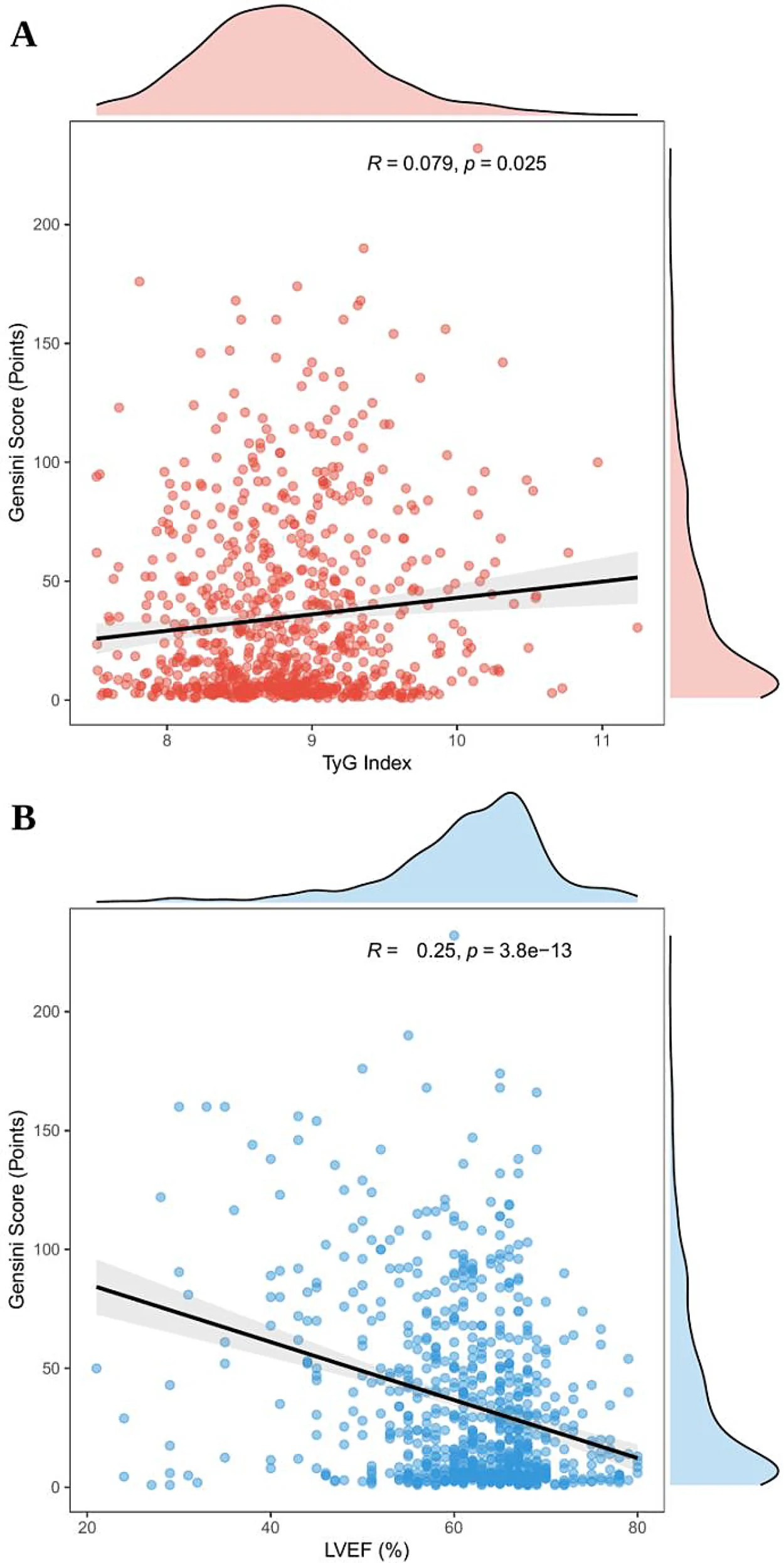
Spearman correlation analyses between metabolic/cardiac functional indicators and coronary lesion severity assessed by the gensini score. **(A)** TyG index showed a weak positive correlation with Gensini score (r = 0.079, *P* = 0.025). **(B)** LVEF displayed an inverse correlation with Gensini score (r = −0.25, *P* < 0.001). Scatter plots include fitted trend lines with 95% confidence bands; marginal density plots depict the distributions of TyG/LVEF and Gensini scores. Adjustment was made for all covariates, including age, sex, and BMI; smoking status and alcohol consumption; and comorbidities of hypertension (HTN), hyperlipidaemia (HLP), and type 2 diabetes mellitus (T2DM).

**Figure 6:**
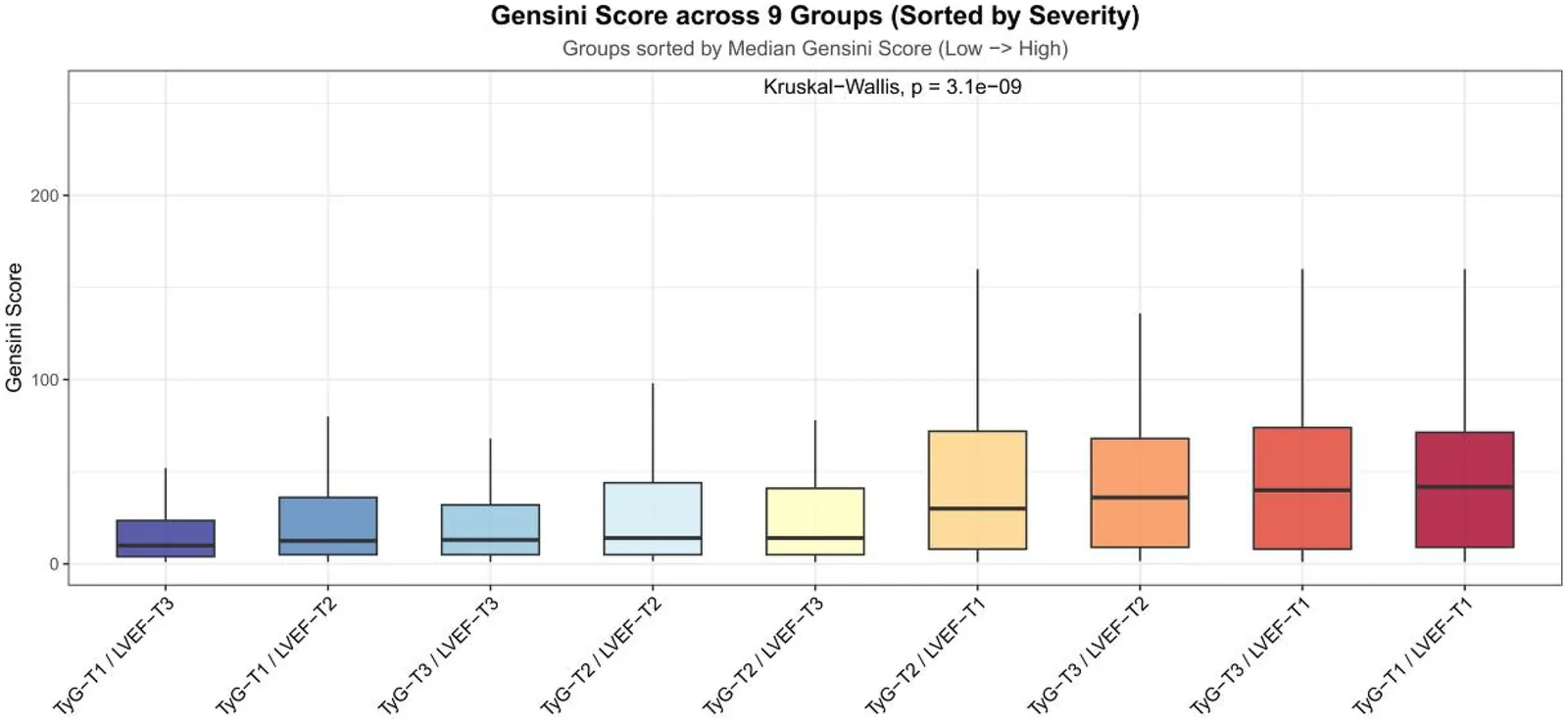
Distribution of coronary lesion severity (gensini score) across nine joint TyG–LVEF categories defined by tertiles (TyG: T1–T3; LVEF: T1–T3). Boxplots are ordered by the median Gensini score from low to high. Overall differences among the nine groups were assessed using the Kruskal–Wallis test (*P* = 3.1 × 10⁻⁹). Boxes represent the interquartile range (IQR) with the median shown as the central line; whiskers indicate the data spread beyond the IQR. Adjustment was made for all covariates, including age, sex, and BMI; smoking status and alcohol consumption; and comorbidities of hypertension (HTN), hyperlipidaemia (HLP), and type 2 diabetes mellitus (T2DM).

Regarding the relationship between composite indices and angiographic disease severity, Spearman correlation showed a weak positive association between the Gensini score and the TyG index (r = 0.079, *P* = 0.025), suggesting that higher TyG levels are linked to a modest increase in coronary lesion burden. In parallel, Gensini scores were inversely correlated with LVEF (r = −0.25, *P* < 0.001), indicating more severe coronary disease among individuals with poorer cardiac function (Figure 5). Quantile-based cross-classification further demonstrated pronounced differences in Gensini scores across the nine groups (Kruskal–Wallis test *P* < 0.001), with a clear graded distribution (Figure 6 and Supplementary Table S5). The TyG-T1/LVEF-T3 group (low metabolic risk/high cardiac function) had the lowest lesion severity, with a median Gensini score of 10.0 (0.2–19.8). As LVEF decreased to the T1 stratum, Gensini scores rose substantially across all TyG categories, reaching medians of 41.8 and 40.0 in the TyG-T1/LVEF-T1 and TyG-T3/LVEF-T1 groups, respectively. Overall, within each LVEF stratum, higher TyG tended to correspond to higher Gensini scores; conversely, within each TyG category, lower LVEF was associated with higher scores. These findings suggest a potentially adverse additive pattern whereby elevated TyG and reduced LVEF jointly relate to a heavier coronary atherosclerotic burden.

## Discussion

In this real-world study of 1,061 symptomatic patients undergoing coronary angiography, the TyG index and LVEF emerged as the most informative independent correlates of CHD. Importantly, the central contribution of our findings extends beyond these individual associations, highlighting the incremental value of jointly integrating “upstream” metabolic stress (TyG) with “downstream” cardiac functional phenotype (LVEF). Adding the triglyceride–glucose index and left ventricular ejection fraction to the base clinical model increased the AUC from 0.655 to 0.694; however, the absolute gain was limited, and overall discrimination remained moderate. Participants with concurrent high TyG and low LVEF had the highest observed probability of coronary heart disease and the highest Gensini scores. Decision-curve analysis based on repeated cross-validation indicated only a modest incremental net benefit. Accordingly, the combined assessment of TyG and LVEF should remain exploratory pending validation in independent populations.

Systemic metabolic dysregulation is a critical driver of CHD pathogenesis, with insulin resistance (IR) acting as the key mechanistic link (20). Our findings support the use of the TyG index as a superior surrogate marker for IR, showing a strong linear positive association with CHD risk that exceeds the predictive power of traditional lipid profiles and other composite metabolic indices, such as AIP and METS-IR. This aligns with recent epidemiological studies highlighting TyG's ability to capture the “residual metabolic risk,” a significant component often overlooked by conventional LDL-C measurements (21, 22). From a mechanistic standpoint, TyG reflects the combined effects of hypertriglyceridemia and hyperglycemia, both of which are central to “glucolipotoxicity,” a condition that promotes atherosclerosis through multiple converging pathways (12). Triglyceride-rich lipoproteins (TRLs) and their remnants contribute to endothelial dysfunction and foam cell formation, while hyperglycemia exacerbates oxidative stress and accelerates the deposition of advanced glycation end-products (AGEs), which in turn amplify vascular inflammation and plaque instability (23). Collectively, these mechanisms lend biological plausibility to the observed association between elevated TyG and CHD. Nevertheless, evidence linking TyG to angiographic disease severity remains inconsistent. In our cohort, TyG was only weakly correlated with the Gensini score (r = 0.079), whereas Özmen et al. reported no significant association between TyG and the SYNTAX score among 214 patients with acute coronary syndrome (*P* = 0.312) (24). These discrepancies may reflect differences in study populations and sample sizes, as well as the distinct dimensions of coronary atherosclerotic burden captured by the Gensini and SYNTAX scoring systems.

As a downstream phenotypic manifestation within the ischemic continuum, alterations in cardiac structure and function represent the cumulative consequences of pathological stress. Our analysis revealed a non-linear inverse relationship between LVEF and CHD risk, highlighting the direct impact of myocardial dysfunction on disease susceptibility. Importantly, even within the range of preserved ejection fraction, lower LVEF values may signal subclinical myocardial stunning or coronary microvascular dysfunction, both of which often precede noticeable angiographic stenosis. This observation further underscores the prognostic value of LVEF as an indicator of functional reserve (25). Metabolic dysregulation may contribute to myocardial injury and remodelling through mechanisms such as glucolipotoxicity, although this pathway cannot be established from the present cross-sectional data. Clinical evidence linking metabolic status to cardiac function remains inconsistent (17, 26). In a retrospective study of 125 individuals with obesity, Özmen et al. reported no significant differences in diastolic function or myocardial performance index between participants with and without metabolic syndrome (27). Such variability suggests that metabolic-functional associations may depend on the population studied, the metabolic phenotype examined, and the echocardiographic measures assessed.

From a translational perspective, TyG and LVEF are inexpensive, routinely available measures of metabolic burden and left ventricular systolic function, respectively, and may complement conventional clinical risk factors. Established long-term cardiovascular risk tools, including SCORE2 and the Framingham score, primarily incorporate traditional clinical variables (28, 29). Previous studies suggest that echocardiographic parameters may refine prognostic stratification in chronic coronary syndrome, while associations between metabolic dysfunction and cardiac phenotypes have received increasing attention (30, 31). In the present study, adding TyG and LVEF to the base clinical model increased the AUC from 0.655 to 0.694, with a comparable gain after repeated cross-validation. Decision-curve analysis indicated greater net benefit for the augmented model across a range of threshold probabilities, although the absolute improvement was modest. Accordingly, joint assessment of TyG and LVEF may offer limited supplementary information beyond existing clinical evaluation, but its incremental clinical value requires further validation.

The coexistence of greater metabolic burden and lower LVEF is biologically plausible, as metabolic disturbances such as lipotoxicity may contribute to myocardial injury and ventricular remodelling (32). Nevertheless, the cross-sectional design precludes establishing the temporal sequence and cannot determine whether metabolic abnormalities directly impair cardiac function or modify treatment response. The high-TyG, low-LVEF phenotype should therefore be regarded as an exploratory clinical profile rather than a marker for treatment selection or response prediction. SGLT2 inhibitors and GLP-1 receptor agonists have demonstrated cardiovascular benefits in selected populations with T2DM and CVD (33). The present findings, however, provide only an epidemiological rationale for testing whether this phenotype is associated with differential treatment benefit. Further prospective interventional studies are required to evaluate this hypothesis.

This study presents several key methodological strengths. First, it leverages a real-world continuous cohort of over 1,000 patients presenting with chest pain, with all diagnoses of CHD and lesion severity confirmed through coronary angiography, the gold standard. This approach minimizes classification bias typically associated with studies relying on ICD codes or self-reported data, thereby ensuring high diagnostic accuracy and robust findings. Second, we adopted a multidimensional assessment framework that integrates the TyG index, representing “upstream” metabolic burden, with LVEF, reflecting “downstream” cardiac functional reserve. This combined approach surpasses the limitations of single-marker analyses, offering a more holistic view of the interplay between metabolic factors and cardiac function in CHD (33). Finally, our study evaluated the exploratory clinical utility of combining routine biochemical tests with non-invasive echocardiography using repeated cross-validated decision-curve analysis. Although this approach showed a modest net-benefit advantage, it should not be considered an effective gatekeeper until externally validated (34).

This study has several limitations. First, the cross-sectional design prevents the establishment of causal relationships, and the use of single-time point measurements may be influenced by short-term fluctuations; therefore, prospective studies are needed to validate these findings. Second, while we have adjusted for major confounding factors, residual confounding from unmeasured genetic or environmental factors remains a possibility. Third, preadmission medication use was not systematically recorded. Statins and other lipid-lowering therapies may have altered lipid-derived indices. Sodium-glucose cotransporter 2 inhibitors and glucagon-like peptide-1 receptor agonists may likewise have influenced metabolic and cardiac parameters, leaving scope for residual confounding and treatment-related bias. Fourth, although the Gensini score quantified overall coronary stenosis burden, specific anatomical features were not assessed separately. These included the number of diseased vessels, left main involvement, chronic total occlusion, calcification, and SYNTAX-defined lesion complexity. Fifth, the augmented model achieved only moderate discrimination, with an apparent AUC of 0.694 and a cross-validated AUC of 0.683. Although exploratory decision-curve analysis indicated a modest net benefit, model calibration, net reclassification improvement, comparison with an established chest-pain score, and external validation were not undertaken. The CHD group exhibited lower BMI and lipid levels, which may reflect the effects of aging, disease-related cachexia (reverse causality), or prior use of lipid-lowering medications (treatment bias), rather than genuinely lower risk. Lastly, the single-center cohort of Asian patients undergoing coronary angiography for chest pain introduces potential selection bias, meaning caution is required when generalizing these findings to the broader population or other ethnic groups.

## Data Availability

The original contributions presented in the study are included in the article/Supplementary material, further inquiries can be directed to the corresponding author.

## Supplementary material

Supplementary Table S1 Adjusted odds ratios for CHD across combined TyG-LVEF tertile groups (reference: High TyG + Low LVEF).

Supplementary Table S2 Subgroup analyses of the association between LVEF and CHD risk across predefined strata.

Supplementary Table S3 Subgroup analyses of the association between TyG and CHD risk across predefined strata.

Supplementary Table S4 Diagnostic performance of TyG, LVEF, the base clinical model (age, sex, BMI, smoking, alcohol consumption, and HTN), and the model augmented with TyG and LVEF for CHD discrimination.

Supplementary Table S5 Distribution of Gensini scores across nine TyG-LVEF tertile-combination groups.
